# Supplementary material for: Influenza A Virus Infection Reactivates Human Endogenous Retroviruses Associated with Modulation of Antiviral Immunity
Source: Viruses. 2022 Jul 21;14(7):1591. doi: 10.3390/v14071591 (PMC9320126; doi:10.3390/v14071591)
Supplement: Supplementary file 1 [file viruses-14-01591-s001.zip › MotifAnalysis.zip!/MotifOutput_SC35MdNS1/homerResults/motif5.similar.html]

motif5

## Information for motif5

A
G
T
C
A
C
G
T
A
C
G
T
C
T
A
G
A
T
C
G
C
G
T
A
T
A
C
G
G
C
T
A
C
A
T
G
G
A
T
C
A
G
T
C
A
G
C
T
  
Reverse Opposite:  

C
T
G
A
C
T
A
G
C
T
A
G
G
T
A
C
C
G
A
T
A
T
G
C
A
C
G
T
A
T
G
C
A
G
T
C
T
G
C
A
T
G
C
A
T
A
C
G
  

|  |  |
| --- | --- |
| p-value: | 1e-57 |
| log p-value: | -1.329e+02 |
| Information Content per bp: | 1.729 |
| Number of Target Sequences with motif | 32.0 |
| Percentage of Target Sequences with motif | 41.56% |
| Number of Background Sequences with motif | 150.7 |
| Percentage of Background Sequences with motif | 0.33% |
| Average Position of motif in Targets | 104.9 +/- 50.0bp |
| Average Position of motif in Background | 106.1 +/- 88.1bp |
| Strand Bias (log2 ratio + to - strand density) | 10.0 |
| Multiplicity (# of sites on avg that occur together) | 1.19 |
| Motif File: | file (matrix) reverse opposite |

### Similar de novo motifs found

|  |  |  |  |  |  |  |  |
| --- | --- | --- | --- | --- | --- | --- | --- |
| Rank | Match Score | Redundant Motif | P-value | log P-value | % of Targets | % of Background | Motif file |
| 1 | 0.773 | C T A G A C T G C G T A A C T G C G T A C T G A A G T C A G T C A C G T A C G T | 1e-46 | -107.991159 | 42.86% | 0.84% | motif file (matrix) |
